# The m6A and immune regulatory gene signature predicts the prognosis and correlates with immune infiltration of head and neck squamous cell carcinoma

**DOI:** 10.1016/j.heliyon.2024.e39758

**Published:** 2024-10-24

**Authors:** Jian Xiao, Wei Li, Guolin Tan, Ru Gao

**Affiliations:** Department of Otolaryngology-Head and Neck Surgery, The Third Xiangya Hospital of Central South University, Changsha, Hunan, 410013, China

**Keywords:** m^6^A, Immune, Head and neck squamous cell carcinoma, Prognosis, Risk score

## Abstract

Recent investigations have underscored the epigenetic modulation of the immune response; however, the interplay between RNA N6-methyladenosine (m^6^A) modification and immunomodulation in head and neck squamous cell carcinoma (HNSC) remains relatively unexplored. To bridge this knowledge gap, we undertook an extensive examination of the potential contributions of m^6^A modification and immunomodulation in HNSC. We amalgamated and deduplicated 27 m^6^A -related genes (m6AGs) and 1342 immune regulation-related genes (IMRGs), resulting in a comprehensive dataset encompassing 1358 genes. This dataset was scrutinized for m^6^A modification and immunomodulatory patterns within HNSC specimens. Employing Cox regression analysis and the Least Absolute Shrinkage and Selection Operator (LASSO) technique, we developed a prognostic risk model for m^6^A regulator-mediated methylation modification and immunomodulation-related differentially expressed genes (m6A&IMRDEGs). Our differential expression analysis delineated 29 m6A&IMRDEGs, and Weighted Gene Co-expression Network Analysis (WGCNA) elucidated two module genes (IL11 and MMP13) subjected to correlation analysis. The prognostic prediction models revealed that the clinical predictive efficacy peaked for 1-year forecasts, followed sequentially by 3-year and 5-year predictions. The risk scores derived from the model adeptly categorized HNSC patients into high- and low-risk cohorts, with the high-risk group exhibiting a more unfavorable prognosis. Protein-Protein Interaction (PPI) analysis identified 7 hub genes implicated in m^6^A and immune regulation, namely BPIFB1, BPIFB2, GP2, MUC5B, MUC7, PIP, and SCGB3A1. Furthermore, we noted marked disparities in the expression profiles of 18 immune cell types between the high- and low-risk groups. Our results substantiate that the clustering subpopulations and risk models associated with m^6^A and immune regulatory genes portend a poor prognosis in HNSC. The risk score emerges as a potent prognostic biomarker and predictive metric for HNSC patients. A thorough assessment of m^6^A and immune regulatory genes in HNSC will augment our comprehension of the tumor immune microenvironment and facilitate the advancement of HNSC therapeutics.

## 1 Introduction

Head and neck squamous cell carcinoma (HNSC) ranks as the sixth most prevalent malignancy globally [1]. Despite a modest enhancement in HNSC survival rates over the last three decades, the condition exerts a profound toll, significantly affecting patients' physical and mental well-being [2]. Traditional diagnosis and therapeutic approaches pose challenges in early disease detection and prognosis enhancement [3]. Therefore, the advancement of novel diagnostic biomarkers and prognostic models is imperative for improving the clinical outcomes of HNSC patients.

The N-6-methyladenosine (m6A) modification, initially identified in the 1970s, is the most prevalent and highly conserved mRNA modification in most eukaryotes [4]. Mediated by m6A-binding proteins (readers), demethylases (erasers), and methyltransferases (writers), the m6A modification regulates various aspects of mRNA, including its structure, maturation, stability, splicing, export, translation, and decay [5]. Additionally, m^6^A plays a pivotal role in the regulation of the cell cycle and cellular differentiation [6]. Numerous studies have indicated that m6A-related genes play a role in the progression of both tumor and non-tumor lesions, such as liver hepatocellular carcinoma [7], lung adenocarcinoma [8], pancreatic cancer [9], osteoporosis [10], and acute aortic dissection [11]. In this study, our objective was to investigate the prognostic value of m6A-related hub genes in HNSC.

Traditionally, tumor progression has been attributed to genetic and epigenetic alterations occurring solely within tumor cells. However, extensive research has shown that the tumor microenvironment (TME), consisting of immune and non-immune cells as well as extracellular components, plays a crucial role in tumor recurrence and metastasis [3,12]. Immune checkpoint inhibitors, such as pembrolizumab and nivolumab, have been approved by the FDA for treating recurrent or metastatic HNSC, with pembrolizumab being the primary treatment option for unresectable disease [13]. Although these agents have demonstrated clinical efficacy in a limited subset of patients exhibiting durable responses, the broader patient population experiences marginal or negligible clinical benefit, thereby underscoring a critical unmet clinical need. Consequently, there is an imperative to identify prospective biomarkers and innovative therapeutic targets through an exhaustive analysis of the intricacies of immunomodulation in HNSC.

In recent years, multiple studies have revealed a unique relationship between m^6^A modification and immunomodulation. This interplay includes the modification of T cell proliferation and differentiation, regulation of turnover of essential host transcripts, and methylated viral transcripts to facilitate evasion of the host immune system [14]. m^6^A methylation regulates TME reprogramming and influences the immune escape of cancer cells through various mechanisms, leading to rapid proliferation and evasion of host immune surveillance [15]. Altered TME exhibits characteristics of increased oxygen consumption, nutrient deprivation, accumulation of metabolic byproducts, and immune dysregulation, resulting in a hypoxic, metabolically reprogrammed, and immunosuppressive extracellular niche that promotes tumor immune escape [16]. This phenomenon cannot be solely attributed to RNA degradation mechanisms. Therefore, there is a need to comprehensively analyze the roles of m6A and immunomodulation in HNSC, which would also assist us in further identifying distinct tumor immune phenotypes and enhancing our guidance and predictive capacity for immune therapeutic responses.

This study utilized The Cancer Genome Atlas (TCGA) database to investigate the biological functions and networks associated with methylation modification mediated by m6A regulators and differentially expressed genes related to immunomodulation (M6A&IMRDEGs), aiming to elucidate the role of M6A&IMRDEGs in the prognosis of HNSC patients. A risk model and nomogram were constructed based on M6A&IMRDEGs to predict the prognosis of HNSC patients.

## 2 Materials and methods

### 2.1 Data download

A total of 502 head and neck squamous cell carcinoma (HNSC) samples with clinical information and 44 control samples were obtained after excluding samples lacking clinical information [17]. The sequencing data was converted into FPKM format [18]. For validation, the GSE65858 and GSE83519 datasets [19] related to HNSC were downloaded from the GEO database using the R package GEOquery [20]. Dataset GSE65858 consisted of 270 HNSC samples, while dataset GSE83519 included 22 HNSC samples and 22 control samples. All HNSC samples and control samples were included in the study [21].

To obtain the m6A Modification-related Genes (m6AGs) set, a search was conducted using “m6A″ as the keyword in published literature [22]. This set comprised 27 m6A modification-related genes. Immunomodulation-related genes (IMRGs) were collected from the GeneCards database, specifically retaining “Protein Coding” immune regulation-related genes using “Immunomodulation” as the search term. A total of 1341 IMRGs were obtained. Additionally, a search for immune regulation-related genes in published literature [22] resulted in two additional IMRGs after merging and removing duplicates, resulting in a total of 1342 IMRGs. The merged and deduplicated set of 27 m6A modification-related genes (m6AGs) and 1342 immune regulation-related genes (IMRGs) yielded a total of 1358 genes.

### 2.2 m6A and immune regulation-related differentially expressed genes and functional similarity analysis

The samples obtained from the TCGA-HNSC dataset were categorized into two distinct groups: the HNSC group and the control group. To identify genes that exhibited differential expression, the R package DESeq2 was employed for conducting differential analysis. The criteria utilized to define differentially expressed genes (DEGs) were as follows: |logFC| > 4.0 and adj. p < 0.05. For the up-regulated DEGs, logFC values exceeding 4.0 and adj. p-values below 0.05 were considered significant. Conversely, down-regulated DEGs were defined as having logFC values below −4.0 and adj. p-values below 0.05.

To identify m6A Regulators Mediated Methylation Modification and Immunomodulation-Related Differentially Expressed Genes (m6A&IMRDEGs) associated with HNSC, The DEGs were intersected with integrated m6A modification-related genes (m6AGs) and immune regulation-related genes (IMRGs) to identify m6A Regulators Mediated Methylation Modification and Immunomodulation-Related Differentially Expressed Genes (m6A&IMRDEGs). Functional correlation between the m6A&IMRDEGs was calculated using the R package GOSemSim [23]. Functional similarity analysis was performed to evaluate the functional correlation between m6A&IMRDEGs.

### 2.3 Copy number variation (CNV) and somatic mutation (SNP) analysis

To analyze copy number variations (CNV) in HNSC samples, the “Copy Number Segment” data was acquired by utilizing the R package TCGAbiolinks. This dataset was sourced from TCGA and underwent preprocessing using VarScan software. The SNP profile was then visualized with the assistance of the R package maftools [24].

### 2.4 Construction of m6A modification and immune regulation scores (m6A&IMScore) and weighted gene association network analysis (WGCNA)

The calculation of m6A modification and immune regulation scores (m6A&IMScore) was accomplished through the utilization of Single-sample Gene-Set Enrichment Analysis (ssGSEA). The generation of group comparison charts was facilitated by employing the R package ggplot2. To evaluate the diagnostic efficacy of m6A&IMScore in HNSC development, ROC curves were constructed using the R package pROC, and the corresponding area under the curve (AUC) was calculated. Furthermore, Kaplan-Meier (KM) curves were analyzed and plotted based on m6A&IMScore with the assistance of the R package survival [25].

WGCNA was conducted using the R package WGCNA [26] to explore gene co-expression patterns. The correlation between m6A&IMScore and different modules was assessed, and module-characteristic genes were documented. Modules exhibiting |r value| > 0.30 were identified, and the intersection of all genes within these modules with m6A&IMRDEGs was considered the set of module genes.

### 2.5 Expression difference and correlation analysis of module genes

The ROC curve for the module genes in TCGA-HNSC was generated using the R package pROC, and the corresponding area under the ROC curve (AUC) was calculated to evaluate the diagnostic impact of the expression levels of the Module Genes on the occurrence of HNSC [27]. Correlation analysis was performed using the Spearman algorithm to investigate the relationships among the module genes. The expression levels were analyzed, and a correlation scatter plot was created utilizing the R package ggplot2.

### 2.6 Construction of the prognostic risk model

To build a prognostic risk model in the TCGA-HNSC dataset, we employed the R package survival for multivariate Cox regression analysis, focusing on m6A and immune regulation-related differentially expressed genes (m6A&IMRDEGs) to assess their prognostic significance. Initially, variables with p < 0.10 from univariate Cox regression were selected for LASSO regression analysis to mitigate overfitting. The outcomes of univariate Cox regression were depicted in a Forest Plot, illustrating the expression patterns of m6A&IMRDEGs. Subsequently, LASSO regression was conducted using the glmnet package with family = “binomial”, iterating 500 times to enhance model generalization by imposing a penalty term (lambda × slope absolute value). The LASSO results were visualized in a prognostic risk model and variable trajectory diagrams. Following this, multivariate Cox regression was performed on the m6A&IMRDEGs from the LASSO analysis to identify key genes (Key Genes) for the risk model. The multivariate Cox regression results were visualized to display the expression of these key genes. Finally, the risk score (RiskScore) was computed using the risk coefficients from the multivariate Cox regression analysis. The calculation formula for the risk score is as follows: $\text{Risk}Score=\sum_{i}Coefficient\;\left({gene}_{i}\right)*mRNA\;Expression\;\left({gene}_{i}\right)$.

### 2.7 Prognostic analysis of the prognostic risk model for head and neck squamous cell carcinoma

The optimal cutoff value for the prognostic risk model's risk score (RiskScore) was determined using the R package maxstat [28]. This value was employed to stratify the HNSC samples into high-risk (High Risk) and low-risk (Low Risk) groups. To assess the predictive capability of the risk score for survival time, Kaplan-Meier (KM) curve analysis was conducted utilizing the R package survival [25]. Furthermore, univariate and multivariate Cox regression analyses were performed on the risk groups based on the prognostic risk model and clinical information. Variables with a p-value <0.10 from the univariate Cox regression analysis were selected for inclusion in the multivariate Cox regression analysis. Finally, a nomogram, which depicts the functional relationship among multiple independent variables, was developed using the R package rms [29].

### 2.8 Expression difference and correlation analysis of key genes

To examine the differential expression of key genes in HNSC samples from the TCGA-HNSC dataset, a comparison chart was generated based on the expression levels of these genes in both the high-risk and low-risk groups. Similarly, the expression differences of key genes in the combined GEO dataset (Combined Datasets) HNSC samples were investigated between the high-risk (High Risk) and low-risk (Low Risk) groups. The risk score for the combined GEO dataset was calculated by utilizing the risk coefficient obtained from the multivariate Cox regression analysis of the prognostic risk model and the expression levels of key genes. Subsequently, a comparison chart was constructed based on the expression levels of these genes.

Furthermore, a correlation analysis was performed on the expression levels of key genes in HNSC samples to further elucidate their relationships. The results of the correlation analysis were visualized using a correlation chord diagram generated by the R package igraph.

### 2.9 Analysis of differentially expressed genes in high and low-risk groups of head and neck squamous cell carcinoma

Differential analysis was conducted on genes between the high-risk and low-risk groups using the DESeq2 R package 2 [30]. Genes exhibiting |logFC| > 4.0 and adj. p < 0.05 were identified as differentially expressed genes (DEGs) associated with the high- and low-risk groups. The results of the differential analysis were visually presented through a volcano plot, which was generated using the ggplot2 R package, and a heat map, which was created using the heatmap R package.

### 2.10 Gene Ontology (GO) enrichment analysis

The Gene Ontology (GO) enrichment analysis of DEGs associated with the high- and low-risk groups was performed using the clusterProfiler R package [31]. The Benjamini-Hochberg (BH) method was employed for the correction of adj. p values.

### 2.11 Gene set enrichment analysis (GSEA)

The TCGA-HNSC data were analyzed using the clusterProfiler R package based on the logFC value. The gene set enrichment analysis (GSEA) was performed with the following parameters: the seed was set as 2020, 1000 calculations were conducted, and each gene set (A) contained a minimum of 10 genes and a maximum of 500 genes. The gene set c2.cp.all.v2022.1.Hs.symbols.GMT [All Canonical Pathways] (3050) was obtained from the Molecular Signatures Database (MSigDB) database [32]. The screening criteria for GSEA included adj. p < 0.05 and FDR value < 0.25.

### 2.12 Protein-protein interaction (PPI), construction of the regulatory network, and ESTIMATE analysis

The DEGs associated with the high and low-risk groups were utilized to construct the DEGs-related PPI Network, with a threshold set at a minimum required interaction score of medium confidence (0.400). An mRNA-miRNA Regulatory Network was created 34 to analyze the relationship between m6A and immune regulation-related hub genes and miRNA [33].

The immune and stromal scores of HNSC samples were calculated using the R package IOBR [34]. The ESTIMATE algorithm was employed to quantify the immune score and stromal score based on the expression matrix of HNSC samples, resulting in the determination of the ESTIMATE score. To illustrate the expression differences between the high- and low-risk groups, a group comparison graph depicting three distinct immune infiltration assays was generated using the R package ggplot2.

### 2.13 Immune infiltration assay

The relative abundance of immune cell infiltration in the samples was represented by the enrichment fraction obtained from ssGSEA analysis. Samples with a p-value <0.05 were filtered to create the immune cell infiltration matrix. Subsequently, an R package heatmap was utilized to generate a correlation heatmap.

### 2.14 Statistical analysis

All data processing and analysis in this article were conducted using R software (Version 4.2.0). Continuous variables are presented as mean ± standard deviation. The comparison between the two groups was performed using the Wilcoxon Rank Sum Test. Unless otherwise specified, the results represent correlation coefficients between different molecules calculated through Spearman correlation analysis. A p-value <0.05 was considered statistically significant for all results.

## 3 Results

### 3.1 m6A and immune regulation-related differentially expressed genes and functional similarity analysis

**Fig. 1:**
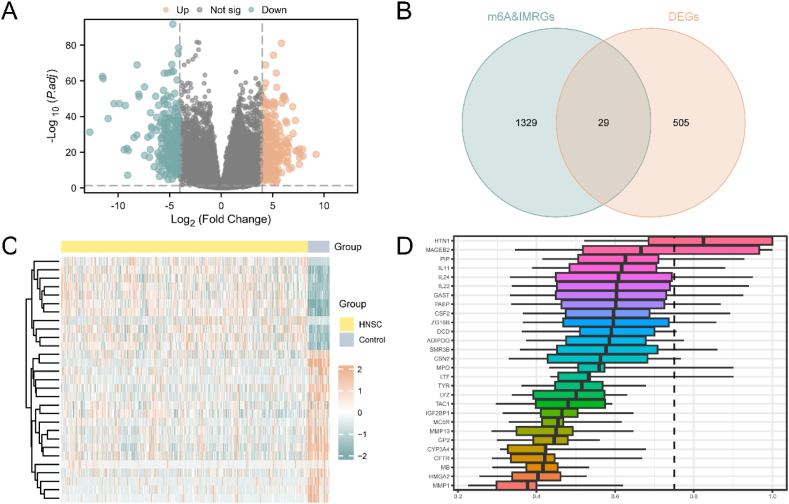
TCGA-HNSC differential Gene expression and Friends analysis. A: The volcano plot displays the DEGs, comparing the HNSC group and the control group. B: The Venn diagram illustrates the intersection of DEGs, integrated m6AGs, and IMRG. C: A heat map is generated to visualize the correlation between m6A&IMRDEGs. D: Friends analysis of m6A&IMRDEGs are presented as a histogram.

Fig. S1 shows the workflow process. The TCGA-HNSC dataset was divided into HNSC and Control groups. Differential gene expression analysis was conducted using DESeq2 in R, resulting in 534 differentially expressed genes (DEGs) in the HNSC dataset. Among these DEGs, 256 were up-regulated and 278 were down-regulated. The volcano map visualized the analysis results (Fig. 1A). By intersecting DEGs with m6A modification-related genes (m6AGs, Table S1) and immune regulation-related genes (IMRGs, Table S2), 29 m6A&IMRDEGs were identified, including IL11, MPO, HTN1, ZG16B, MMP13, IGF2BP1, HMGA2, MMP1, GAST, PAEP, CYP3A4, LYZ, CFTR, CSF2, IL24, SMR3B, PIP, MC5R, MB, MAGEB2, LTF, TAC1, IL22, CGB5, CSN2, ADIPOQ, DCD, TYR, and GP2 (Fig. 1B). Differential expression of m6A&IMRDEGs between sample groups in the HNSC dataset were analyzed, and a heat map (Fig. 1C) was generated using the R package heatmap. Functional similarity analysis identified HTN1 as a crucial gene in the biological process of HNSC, surpassing the cut-off value (cut-off value = 0.75) (Fig. 1D).

### 3.2 Copy number variation (CNV) and somatic mutation (SNP) analysis

**Fig. 2:**
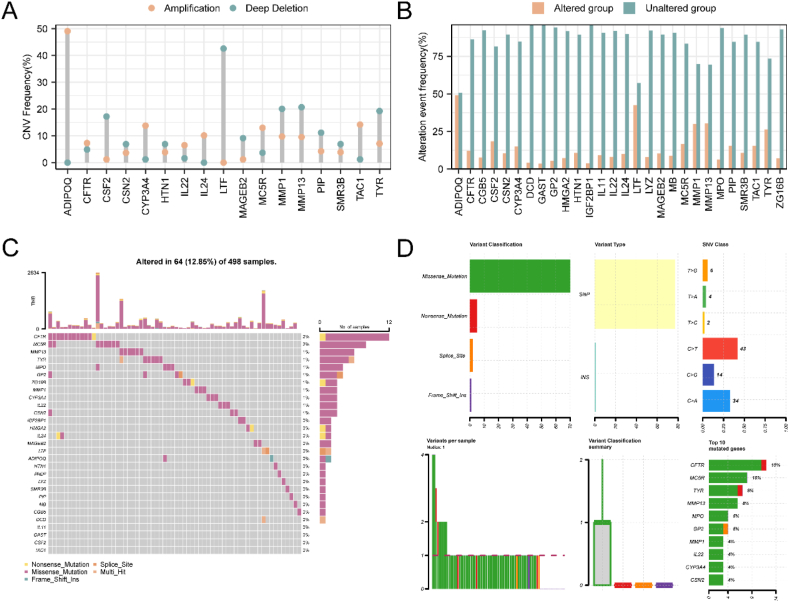
CNV and SNP analysis. A: CNV of m6A&IMRDEGs. B: Alteration Event Frequency analysis of m6A&IMRDEGs. C-D. SNPs of m6A and m6A&IMRDEGs.

To investigate the CNV of 29 m6A&IMRDEG, the R package maftools were employed for analysis and visualization of the results (Fig. 2A). Our findings revealed extensive amplifications and deletions of these genes in HNSC samples. Notably, ADIPOQ exhibited the highest amplification, while LTF displayed the most frequent deletion. Additionally, Alteration Event Frequency analysis demonstrated a higher frequency of copy number alterations for ADIPOQ (Fig. 2B).

Subsequently, we visualized the SNPs of these 29 m6A&IMRDEGs (Fig. 2C). Among them, CFTR exhibited the highest mutation rate. Furthermore, we conducted mutation analysis to visualize the results (Fig. 2D). The analysis revealed five predominant types of somatic mutations, with missense mutations being the predominant type. Moreover, SNPs were the primary mutation type observed for these genes in HNSC samples, with C to T mutation being the most common single nucleotide variant.

### 3.3 Construction of inflammatory programmed cell death score and weighted gene association network analysis (WGCNA)

**Fig. 3:**
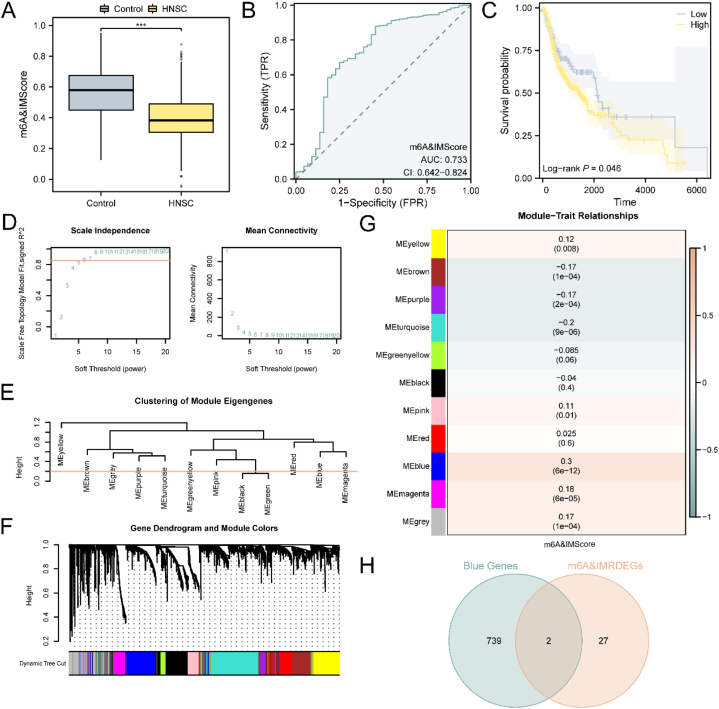
Wgcna for TCGA-HNSC. A. The group comparison boxplot demonstrates the differences in m6A&IMScore between the HNSC and Control groups. B. A ROC curve is plotted to assess the accuracy of m6A&IMScore in the TCGA-HNSC dataset. C. Prognostic KM curves compare the high and low groups of m6A&IMScore in HNSC samples. D. The WGCNA analysis reveals the scale-free network display of the optimal soft threshold. E. Module aggregation results are displayed for the top 10 % of genes with the highest variance. F. The clustering results show the relationship between the top 10 % of genes and the merged module. G. The correlation analysis displays the relationship between the top 10 % gene clustering modules and m6A&IMScore. H. A Venn diagram illustrates the overlap between 29 m6A&IMRDEGs and the MEblue module. ∗∗∗p < 0.001.

In accordance with the expression of 29 m6A&IMRDEGs in the TCGA-HNSC dataset, the m6A modification and immune regulation scores (m6A&IMScores) were calculated for all samples. A comparison of group box plots (Fig. 3A) demonstrated a highly significant difference in m6A&IMScores between the HNSC group and the control group. Additionally, a ROC curve was generated based on the expression of m6A&IMScores in the TCGA-HNSC dataset, revealing modest discriminatory accuracy among different groups (0.5 < AUC <0.7) (Fig. 3B). Moreover, prognostic Kaplan-Meier (KM) curve analysis was conducted by integrating m6A&IMScores with overall survival (OS) of HNSC samples, indicating that the high m6A&IMScores group exhibited a poorer prognosis (Fig. 3C).

To identify co-expression modules within the TCGA-HNSC dataset, WGCNA was performed on the top 10 % of genes with the highest variance. By calculating the scale-free fitting index at various soft thresholds, an optimal soft threshold of 6 was established for constructing the co-expression network (Fig. 3D). The association between the genes and the merged module was visualized using a clustering tree (Fig. 3E). Subsequently, the top 10 % of genes in terms of variance were clustered, and the relationship between the genes and the merged module was further visualized (Fig. 3F). Furthermore, we obtained the correlations between the 11 module genes and m6A&IMScores of the TCGA-HNSC samples (Fig. 3G). Using a correlation threshold of |r value| > 0.30, we identified the module MEblue as being significantly correlated (|r value| = 0.30), and subsequently screened for genes within this module for further analysis. By intersecting the 29 m6A&IMRDEGs with the genes in all screening modules, we identified 2 module genes: IL11 and MMP13 (Fig. 3H).

### 3.4 Expression difference and correlation analysis of module genes

**Fig. 4:**
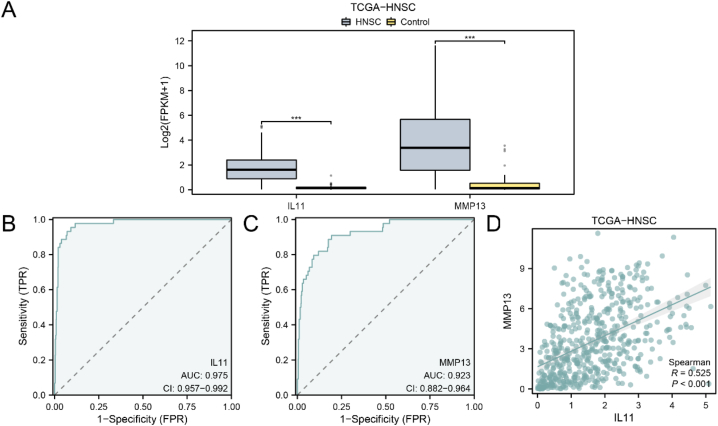
Expression difference analysis for module genes. A. Group comparison boxplot of Module Genes in TCGA-HNSC. B.ROC curve of IL11. B. ROC curve of MMP13. D. Scatter plot showing the correlation between IL11 and MMP13. ∗∗∗p < 0.001.

To examine the expression differences of module genes (Module Genes) in the TCGA-HNSC dataset, a group comparison box plot was employed to analyze their expression levels in the HNSC and control groups (Fig. 4A). The results revealed a highly significant difference in the expression levels of two module genes, namely IL11 and MMP13, between the HNSC and control groups. Next, the R package pROC was used to draw the ROC curve based on the expression levels of the two module genes TCGA-HNSC data set. ROC curves were generated, demonstrating the high accuracy (AUC >0.9) of IL11 and MMP13 expression levels in distinguishing different groups (Fig. 4B–C). Additionally, a scatter plot illustrated a moderate positive correlation (r = 0.525) between IL11 and MMP13 in the integrated GEO datasets (Combined Datasets) (Fig. 4D).

### 3.5 Construction of the prognostic risk model for head and neck squamous cell carcinoma

**Fig. 5:**
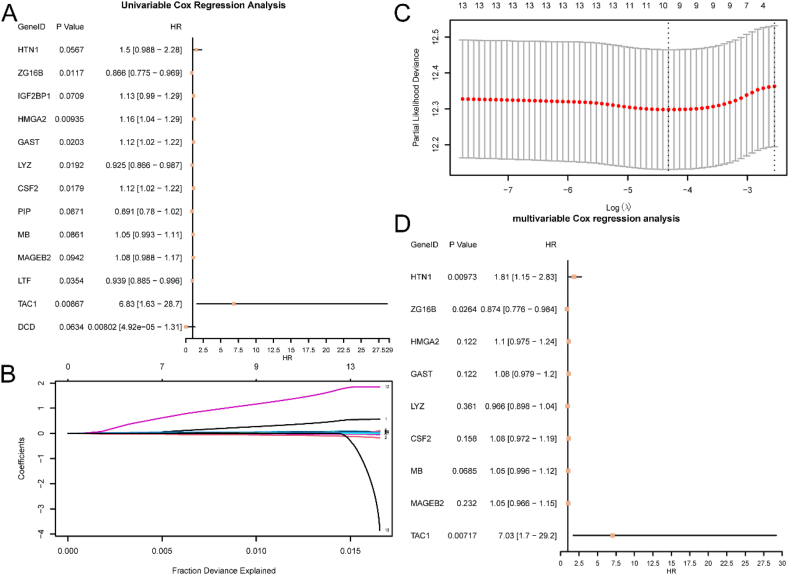
Lasso and Cox regression analysis. A. Forest Plot of 29 m6A&IMRDEGs in the single factor Cox regression model. B-C. Prognostic risk model plot (B) and variable trajectory plot (C) of the LASSO regression model. D. The forest plot of 9 key genes in the multivariate Cox regression model.

To construct a prognostic risk model for HNSC, a univariate Cox regression analysis was conducted using 29 m6A&IMRDEGs. The variables identified in the univariate analysis were visualized using a Forest Plot (Fig. 5A). This analysis revealed statistical significance in 13 m6A&IMRDEGs, including HTN1, ZG16B, IGF2BP1, HMGA2, GAST, LYZ, CSF2, PIP, MB, MAGEB2, LTF, TAC1, and DCD.

To further assess the prognostic value of these m6A&IMRDEGs in the HNSC risk model, a LASSO regression analysis was performed, leading to the construction of a LASSO regression model. The results were visually presented through a LASSO regression model diagram (Fig. 5B) and a LASSO variable trajectory diagram (Fig. 5C). This analysis revealed that the LASSO regression model incorporated 9 key genes: HTN1, ZG16B, HMGA2, GAST, LYZ, CSF2, MB, MAGEB2, and TAC1. Furthermore, a multivariate Cox regression analysis was performed based on these 9 key genes to explore the correlation and prognostic ability between the expression level of the RiskScore and clinical outcome (Fig. 5D).

Additionally, the expression differences of the key genes in HNSC samples from two datasets, namely TCGA-HNSC and the integrated GEO dataset, were investigated. The batch effect of the samples in the HNSC dataset was eliminated after the batch removal process (Fig. S2). In both the TCGA-HNSC dataset and the integrated GEO dataset, statistically significant differences in the expression levels of HMGA2, GAST, LYZ, CSF2, MB, and MAGEB2 were observed between the two groups. Furthermore, correlation analysis was performed on the expression matrix of these nine key genes in both datasets. Notably, a significant positive correlation between HMGA2 and CSF2 was observed in both datasets, while HMGA2 and LYZ showed a significant negative correlation (Fig. S3).

### 3.6 Prognostic analysis of the prognostic risk model for head and neck squamous cell carcinoma

**Fig. 6:**
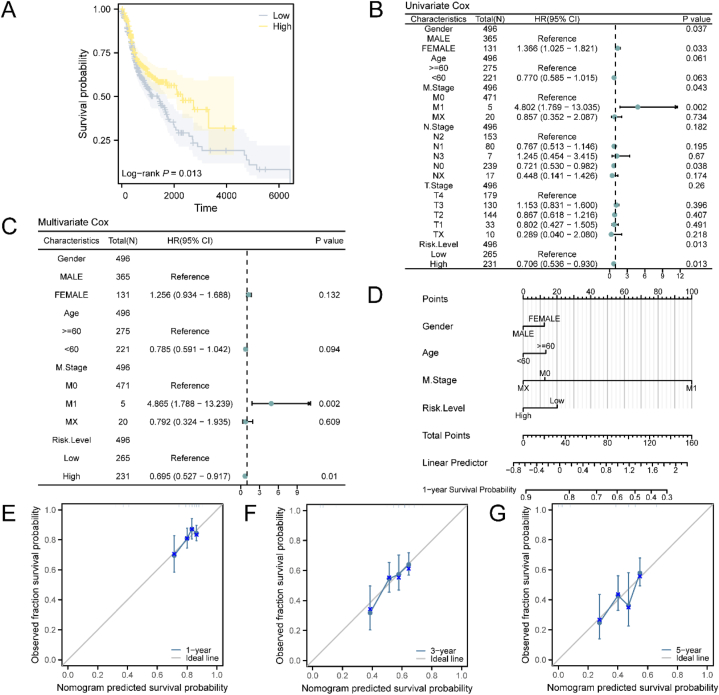
Prognostic analysis. A. The prognostic Kaplan-Meier curve was plotted to compare the high-risk and low-risk groups based on the risk score. B-C. Both univariate Cox regression model (B) and multivariate Cox regression model (C) were employed to analyze the prognostic factors. D. A nomogram was constructed to illustrate the relationship between the risk score and clinical information in the multivariate Cox regression model. E-G. Calibration curves at 1-year (E), 3-year (F), and 5-year (G) intervals were generated to assess the performance of the prognostic risk model.

A prognostic analysis of the prognostic risk model for HNSC was conducted. Kaplan-Meier curve analysis, based on the RiskScore, demonstrated that the high-risk group exhibited a less favorable prognosis compared to the low-risk group (Fig. 6A). Statistically significant associations were observed between the risk level and three clinical factors (Gender, Age, and M Stage) in both univariate and multivariate Cox regression analyses (Fig. 6B–C). The nomogram depicted the correlations among the risk level, clinical factors, and HNSC samples (Fig. 6D). Importantly, M Stage demonstrated higher utility in the prognostic risk model than other variables, while Gender exhibited lower utility. Furthermore, we conducted prognostic calibration analysis at 1-year (Fig. 6E), 3-year (Fig. 6F), and 5-year (Fig. 6G) intervals using the HNSC prognostic risk model, generating calibration curves. The results indicated that the prognostic risk model exhibited superior clinical predictive effectiveness at 1 year >3 years >5 years.

### 3.7 Analysis of differentially expressed genes in high and low-risk groups of head and neck squamous cell carcinoma

**Fig. 7:**
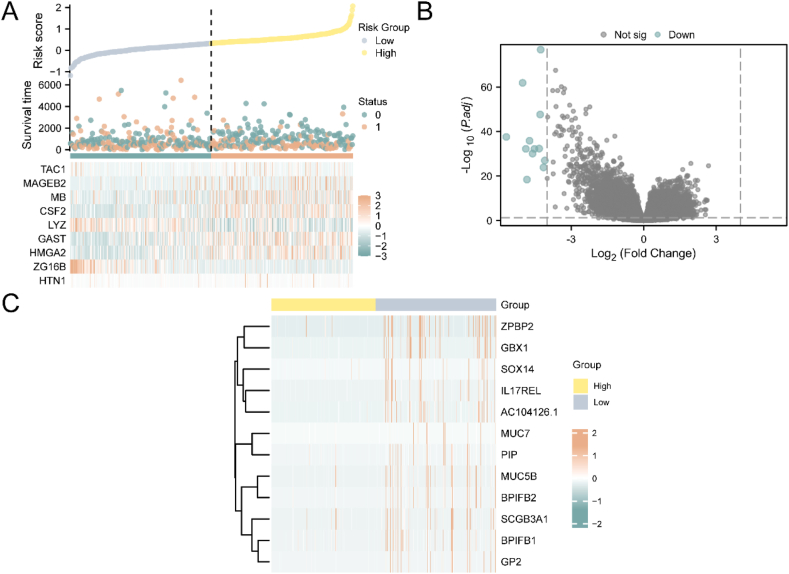
Risk group analysis. A. Risk factor plot for the prognostic risk model for HNSC. B. Volcano plot of differentially expressed genes in the high-risk group and low-risk group in HNSC samples. C. Heat map of DEGs.

The high-risk and low-risk groups, delineated by an optimal cutoff value, were presented in Fig. 7A. A dot plot was used to visualize the survival time and outcome of clinical samples. Differential analysis was conducted between the high-risk and low-risk groups, resulting in the identification of 12 down-regulated genes (IL17REL, MUC5B, SCGB3A1, BPIFB2, AC104126.1, BPIFB1, ZPBP2, MUC7, GBX1, PIP, GP2, SOX14) that satisfied the threshold |logFC| > 4.0 and adj. p < 0.05 (Fig. 7B). Furthermore, a heat map was created to depict the expression of 9 key genes in HNSC samples (Fig. 7C).

The GO enrichment analysis revealed that the 12 differentially expressed genes primarily participated in biological processes such as mucosal immune response and organ/tissue-specific immune response in HNSC. Additionally, they were enriched in cellular components such as Golgi lumen, zymogen granule, membrane, anchored component of the external side of the plasma membrane, and intrinsic component of the external side of the plasma membrane. These genes also exhibited molecular functions such as IgG binding, immunoglobulin binding, aspartic-type endopeptidase activity, and aspartic-type peptidase activity (Fig. S4 and Table S3).

The GSEA analysis demonstrated significant enrichment in biologically relevant functions and signaling pathways, including TGF-beta Receptor Signaling, Negative Regulation of NOTCH4 Signaling, Ion Homeostasis, and Zinc Homeostasis (Fig. S5 and Table S4).

The PPI Network identified 7 hub genes related to m6A and immune regulation, namely BPIFB1, BPIFB2, GP2, MUC5B, MUC7, PIP, and SCGB3A1. Additionally, an mRNA-miRNA regulatory network consisting of these 7 hub genes and 39 miRNAs was constructed. Furthermore, Figure 13C indicated statistically significant differences in the expression levels of ESTIMATE Score and Immune Score between the high-risk and low-risk groups (Fig. S6 and Table S5).

### 3.8 Immune infiltration analysis

**Fig. 8:**
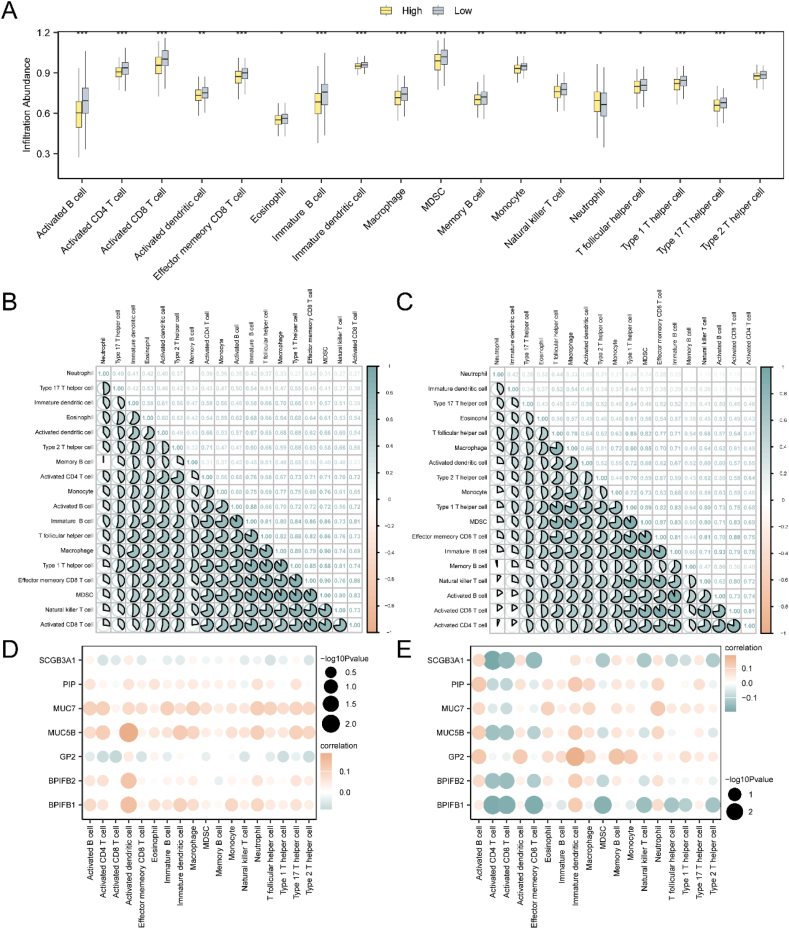
Risk Group Immune Infiltration Analysis by ssGSEA Algorithm. A. boxplot was used to compare the immune cell populations between the high-risk and low-risk groups. B-C. Correlation analysis was conducted to assess the abundance of immune cell infiltration in the high-risk (B) and low-risk (C) groups. D-E. Dot plots were generated to illustrate the correlations between the abundance of immune cell infiltration and the m6A and immune regulation-related hub genes in the high-risk (D) and low-risk (E) groups. ∗p < 0.05, ∗∗p < 0.01, and ∗∗∗p < 0.001.

Significant differences were observed in the expression levels of various immune cells between the high-risk and low-risk groups, including Activated B cell, Activated CD4 T cell, Activated CD8 T cell, Effector memory CD8 T cell, Immature B cell, Memory B cell, T follicular helper cell, Type 1 T helper cell, Type 17 T helper cell, Type 2 T helper cell, Activated dendritic cell, Eosinophil, Immature dendritic cell, Macrophage, MDSC, Monocyte, Natural killer T cell, and Neutrophil (Fig. 8A). Moreover, negative correlations for all immune cells between the high-risk and low-risk groups of HNSC were revealed by correlation heat maps (Fig. 8B–C). Additionally, correlation dot plots illustrated the relationship between m^6^A and immune regulation-related hub genes and the abundance of immune cell infiltration in the high-risk and low-risk groups of HNSC (Fig. 8D–E). Notably, in the high-risk group, a significant positive correlation was observed between MUC5B and activated dendritic cells, while in the low-risk group, SCGB3A1 showed a significant negative correlation with activated CD4 T cells.

## 4 Discussion

The incidence of HNSC is steadily increasing, with an expected 30 % increase projected by 2030, resulting in approximately 1.08 million new cases annually [35]. Furthermore, survivors of this cancer face a high suicide rate, with psychological distress and a decline in quality of life potentially serving as key underlying factors for suicide [36]. Despite advancements made in the diagnosis and treatment of HNSC patients over the past three decades, there has been limited improvement in patient prognosis and quality of life. Therefore, there is a pressing need for more comprehensive and in-depth research to develop more effective treatment modalities that can ultimately enhance the prognosis and survival quality of individuals with HNSC. Additionally, mounting evidence suggests the crucial roles of m6A modification and immune infiltration in the tumor microenvironment (TME) in tumor progression [37,38]. Investigating the roles of m6A modification and immune regulatory-related genes in the occurrence and development of HNSC contributes to a better understanding of the immunobiology of HNSC, facilitating the identification of predictive biomarkers and the development of more effective immunotherapy strategies.

In this study, 29 m6A & IMRDEGs were delineated via differential expression analysis. Subsequent analyses encompassed GO, CNV, and SNP evaluations. Two Module Genes (IL11 and MMP13) were discerned through WGCNA and underwent correlation analysis. The prognosis of HNSC patients was forecasted utilizing the Cox model and LASSO model, with the clinical predictive efficacy hierarchy established as 1 year >3 years >5 years. HNSC patients were categorized into high- and low-risk groups based on risk scores derived from the prognostic risk model, with the high-risk group manifesting a less favorable prognosis compared to the low-risk group. Differential gene analysis was conducted, followed by GO and GSEA analyses to elucidate gene enrichment patterns. Additionally, PPI analysis pinpointed 7 hub genes implicated in m6A modification and immune regulation, including BPIFB1, BPIFB2, GP2, MUC5B, MUC7, PIP, and SCGB3A1. Significant variations in the expression levels of 18 immune cell types were also observed between the high- and low-risk groups.

To construct a prognostic risk model for HNSC, nine key genes were identified: HTN1, ZG16B, HMGA2, GAST, LYZ, CSF2, MB, MAGEB2, and TAC1. Extensive research has identified a set of genes, including HTN1, HMGA2, GAST, LYZ, CSF2, and MAGEB2, as being implicated in the progression of HNSC. These genes have been reported as biomarkers indicative of HNSC development and progression. Additionally, ZG16B, HMGA2, and CSF2 have been associated not only with the disease progression but also with therapeutic responses in HNSC, suggesting their potential roles in treatment stratification and personalized medicine approaches for this malignancy [[39], [40], [41], [42], [43], [44]]. Furthermore, the RiskScore, constructed using HTN1, ZG16B, HMGA2, GAST, LYZ, CSF2, MB, MAGEB2, and TAC1, is correlated with HNSC patient gender, age, and M stage and serves as an independent risk factor for poor prognosis in HNSC patients. Preliminary evidence suggests that M6A&IMRDEGs play important biological roles in the progression of HNSC and can be utilized to determine the prognosis and survival of HNSC patients.

Additionally, differentially expressed genes (DEGs) between the high-risk group and low-risk group were subjected to GO functional analysis and GSEA analysis. The results revealed that the majority of DEGs identified from the GO functional analysis were associated with the structure of the extracellular matrix (ECM) and immune response, which are critical in the occurrence and development of HNSC. The ECM directly interacts with cells through integrins or other cell surface receptors, regulating various cellular processes such as growth, metabolism, migration, proliferation, and differentiation. It also plays a crucial role as a component of the tumor microenvironment, influencing tumor immunity. The GSEA results demonstrated significant enrichment in signaling pathways including TGF-beta Receptor Signaling, Negative Regulation of NOTCH4 Signaling, Ion Homeostasis, and Zinc Homeostasis, all closely linked to immune regulation [[45], [46], [47], [48], [49]]. Among these pathways, TGF-β signaling is known to play a pivotal role in tumor development and immune suppression within the tumor microenvironment [45]. Ion Homeostasis and Zinc Homeostasis are involved in cancer occurrence and tumor immune regulation [47,48]. In conclusion, these findings highlight the substantial impact of M6A&IMRDEGs on the occurrence and development of HNSC, providing valuable insights for diagnostic implications in HNSC research.

The significance of the immune system in the development and treatment of HNSC has long been acknowledged, leading to early attempts to utilize strategies that activate the body's immunity for treating this type of cancer [3,[50], [51], [52]]. Evidence suggests that the expression level of PDL1 in tumors may indicate the potential for clinical benefit, resulting in the approval of pembrolizumab as a first-line treatment for patients with recurrent or metastatic HNSCC in 2019 [53]. In this study, we observed significant differences between the high-risk group and the low-risk group in terms of various immune cell types, including Activated B cells, Activated CD4 T cells, Activated CD8 T cells, Effector memory CD8 T cells, Immature B cells, Memory B cells, T follicular helper cells, Type 1 T helper cells, Type 17 T helper cells, Type 2 T helper cells, Activated dendritic cells, Eosinophils, Immature dendritic cells, Macrophages, MDSCs, Monocytes, Natural killer T cells, and Neutrophils. Additionally, there was a significant correlation between hub genes in the risk model and immune cell infiltration. These findings suggest that a risk model constructed using key genes of M6A&IMRDEGs can assess the immune status of tumors and predict treatment outcomes, which has important implications. Specifically, in the high-risk group, MUC5B exhibited the most significant positive correlation with Activated dendritic cells, while in the low-risk group, SCGB3A1 showed a significant negative correlation with Activated CD4 T cells. This implies that M6A modification may enhance the expression of MUC5B in Activated dendritic cells, while it may decrease the expression of SCGB3A1 in Activated CD4 T cells.

However, this study has several limitations. Firstly, further validation through experiments was not performed. Secondly, there is a lack of corresponding clinical correlation studies, which hinders the analysis of clinical information. Thirdly, the presence of multiple datasets may introduce batch effects that cannot be completely avoided or removed during the analysis, as well as a limited sample size. Lastly, a larger sample size is required to ensure the reliability and stability of the experiments.

In summary, our study comprehensively investigated the M6A&IMRDEGs in HNSC and developed a relevant prognostic risk model. This model can be used to comprehensively evaluate the m6A modification patterns and corresponding tumor microenvironment cellular infiltration characteristics in HNSC patients, thereby identifying the immune phenotype of the tumor and providing new insights for future personalized cancer immunotherapy. Nevertheless, further validation is necessary to elucidate the specific pathogenic mechanisms and molecular targets involved.

## Supplementary information

Additional file 1: Fig. S1: Technology Roadmap. Fig. S2: Batch Effects Removal of GSE65858 and GSE83519. Fig. S3: Expression Difference and Correlation Analysis for Key Genes. Fig. S4: GO Enrichment Analysis for Risk Group DEGs. Fig. S5: GSEA for Risk Group. Fig. S6: PPI Network, Regulatory Network, and ESTIMATE Analysis.

Additional file 2: Table S1: m6A modification-related genes. Table S2: Immune regulation-related genes. Table S3: Results of GO Enrichment Analysis for Risk Group DEGs. Table S4: Results of GSEA for Risk Group. Table S5: mRNA-miRNA list.

## CRediT authorship contribution statement

**Jian Xiao:** Writing – review & editing, Writing – original draft, Visualization, Methodology, Conceptualization. **Wei Li:** Validation, Supervision, Funding acquisition, Conceptualization. **Guolin Tan:** Validation, Supervision, Conceptualization. **Ru Gao:** Writing – review & editing, Writing – original draft, Visualization, Methodology, Conceptualization.

## Data availability

The datasets used and analyzed during the current study are available from the corresponding author upon reasonable request.

## Ethics approval

The institutional review board of the Third Xiangya Hospital of Central South University in Hunan Province, China, determined that the study did not need approval because it used publicly available data.

## Funding

This work was supported by the 10.13039/501100001809National Natural Science Foundation of China [grant number 81870708].

## Declaration of competing interest

The authors declare that they have no known competing financial interests or personal relationships that could have appeared to influence the work reported in this paper.

## Abbreviations

TCGA: The Cancer Genome Atlas
HNSC: Head and Neck Squamous Cell Carcinomas
DEGs: Differentially Expressed Genes
IMRGs: Immunomodulation-Related Genes
m6AGs: m6A Regulators Mediated Methylation Modification Genes
m6A&IMRDEGs: m6A Regulators Mediated Methylation Modification and Immunomodulation-Related Differentially Expressed Genes
CNV: Copy Number Variations
SNP: Single Nucleotide Polymorphism
m6A&IMScore: m6A Regulators Mediated Methylation Modification & Immunomodulation Score
WGCNA: Weighted Correlation Network Analysis
LASSO: Least Absolute Shrinkage and Selection Operator
GO: Gene Ontology
GSEA: Gene Set Enrichment Analysis
