# Supplementary figures and images for: The m6A and immune regulatory gene signature predicts the prognosis and correlates with immune infiltration of head and neck squamous cell carcinoma
Source: Heliyon. 2024 Oct 24;10(21):e39758. doi: 10.1016/j.heliyon.2024.e39758 (PMC11550037; doi:10.1016/j.heliyon.2024.e39758)

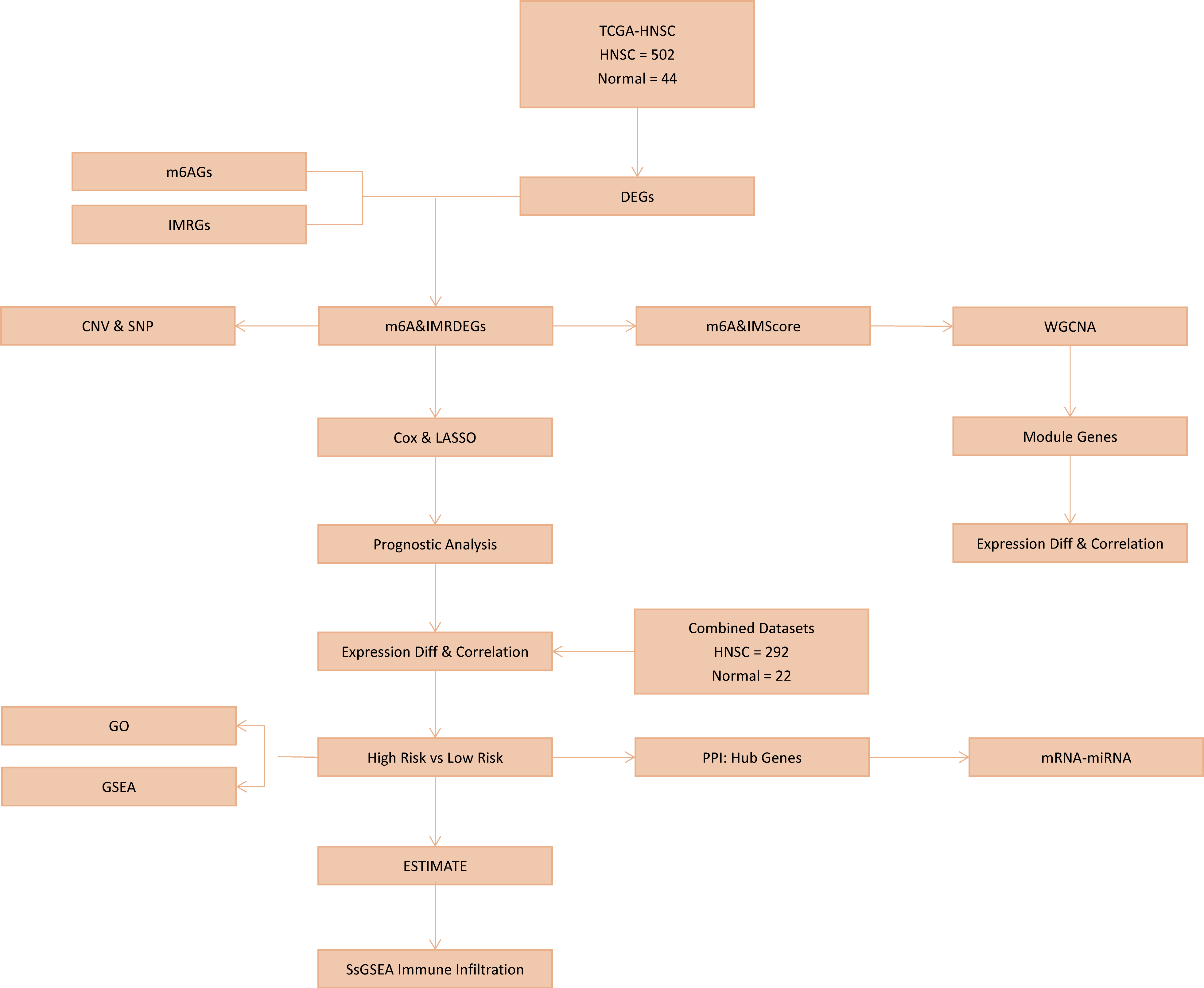

Supplement: Multimedia component 1 [file mmc1.zip › Supplementary Files/Figure S1.tif]

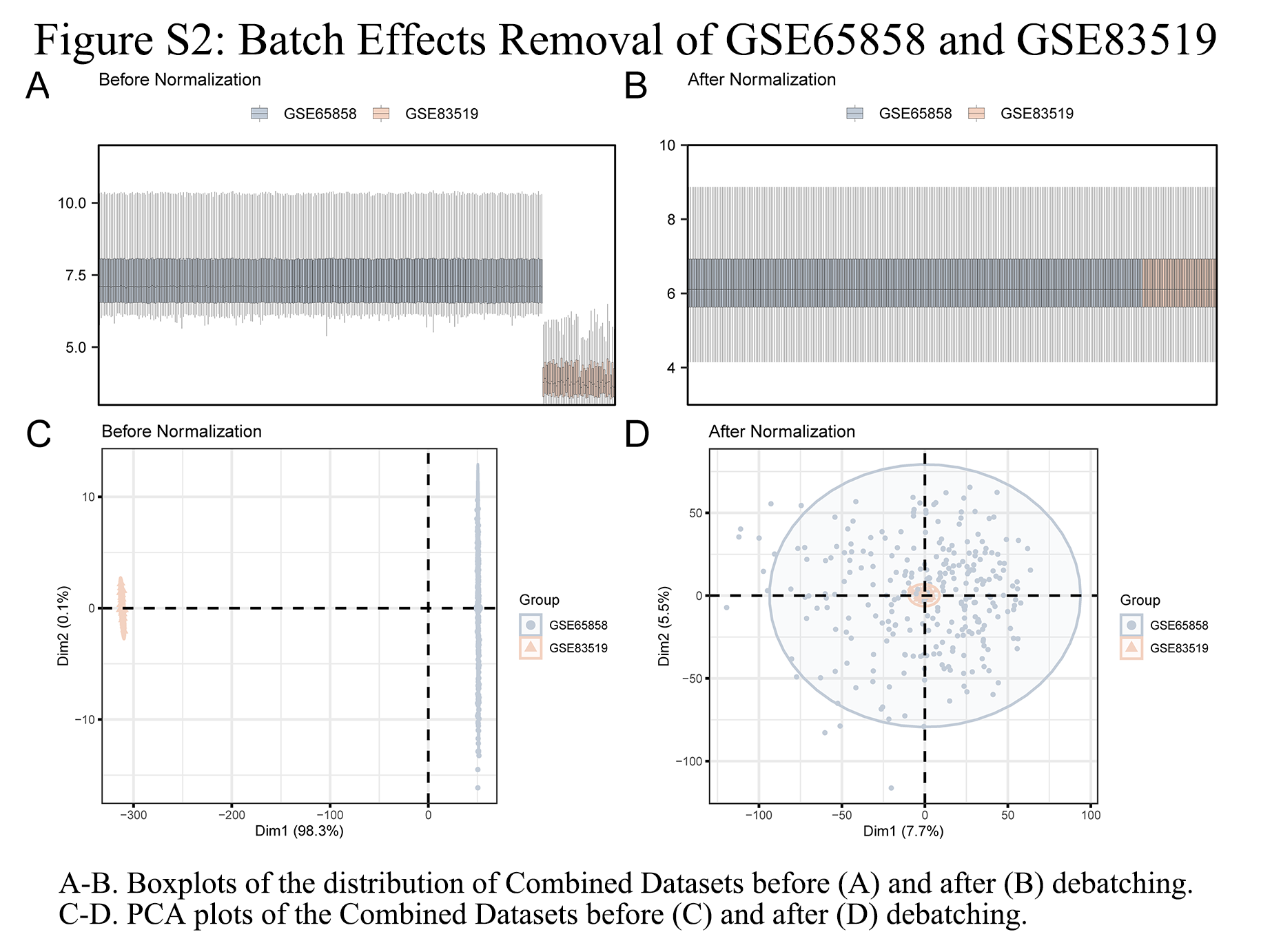

Supplement: Multimedia component 1 [file mmc1.zip › Supplementary Files/Figure S2.tif]

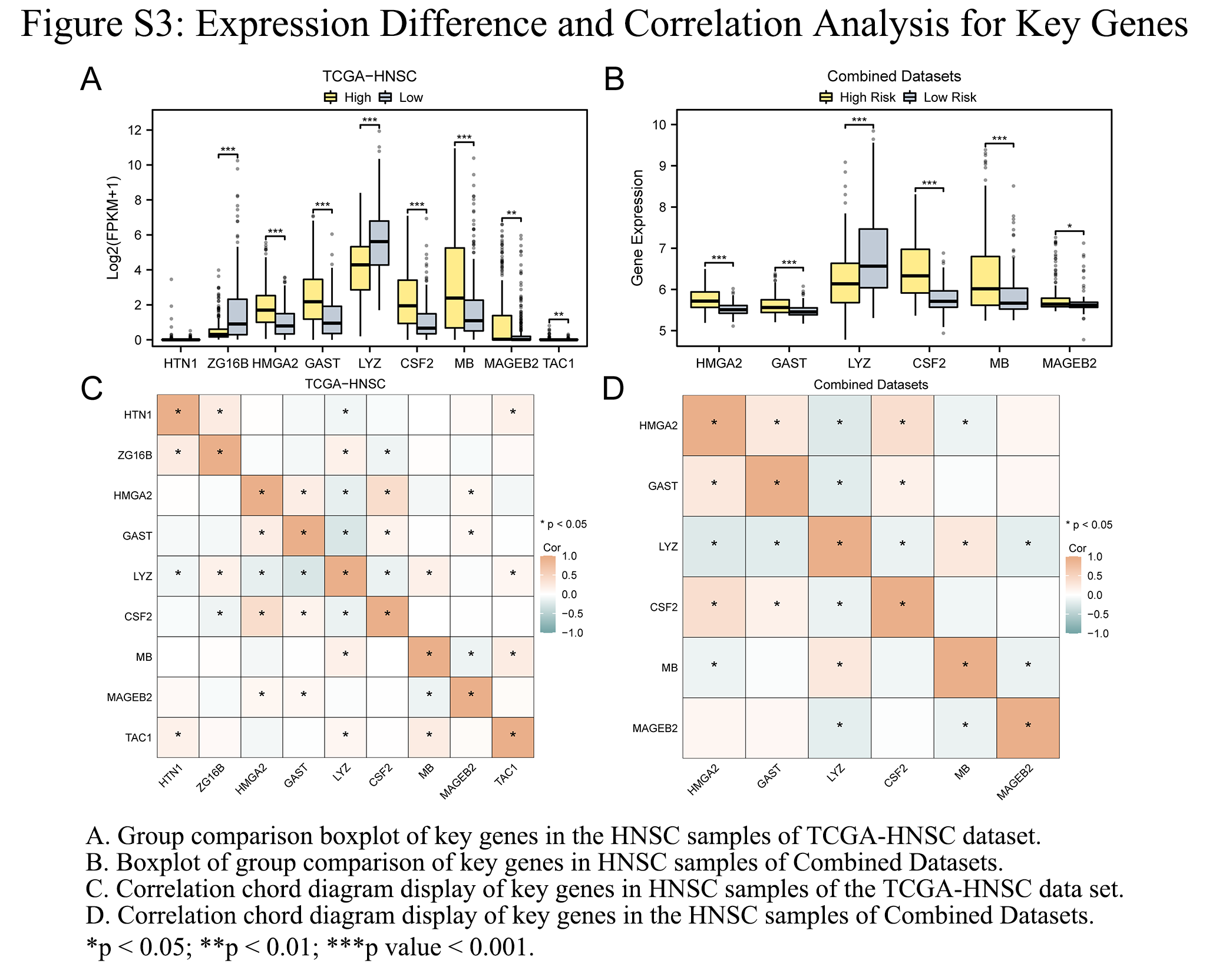

Supplement: Multimedia component 1 [file mmc1.zip › Supplementary Files/Figure S3.tif]

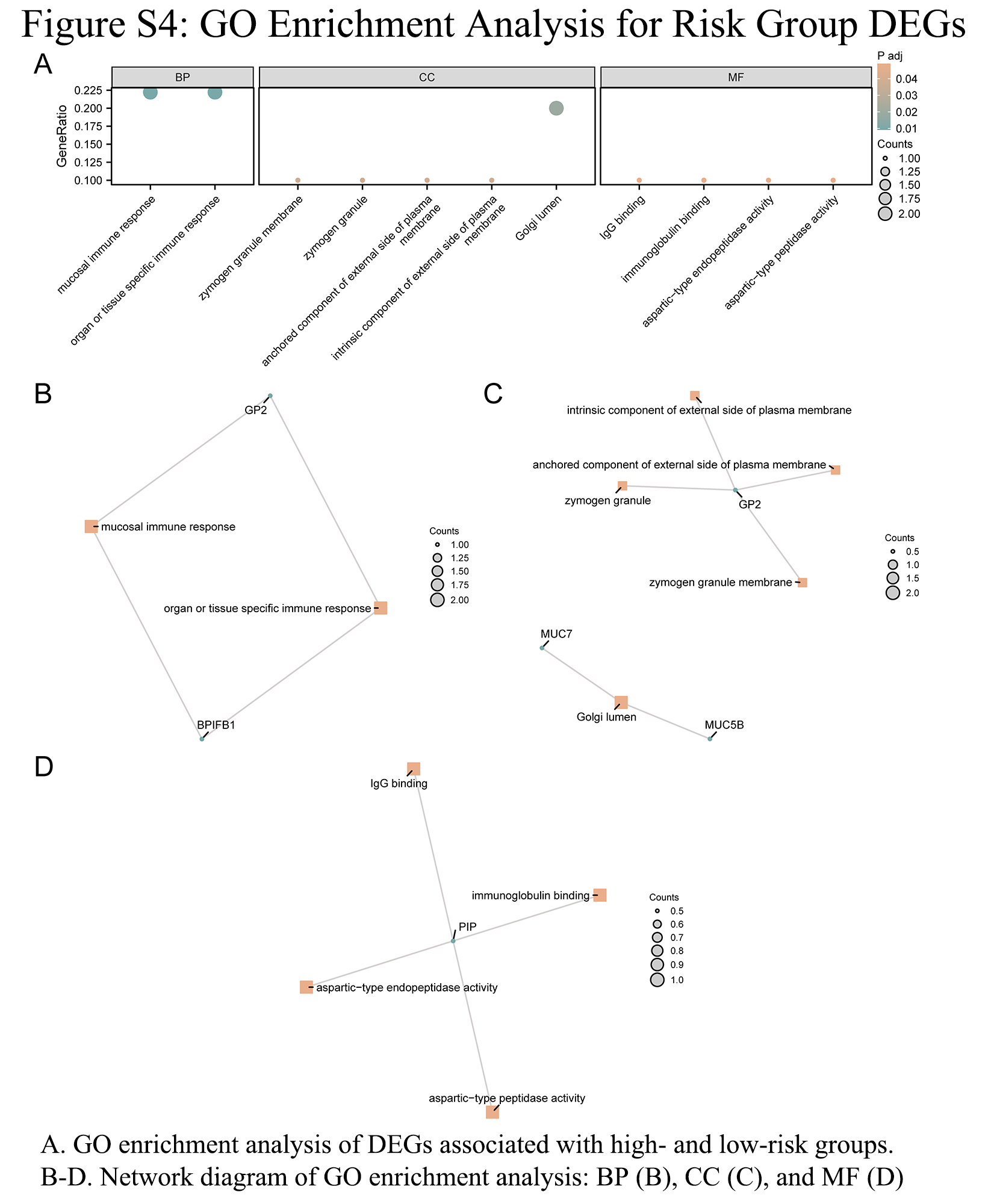

Supplement: Multimedia component 1 [file mmc1.zip › Supplementary Files/Figure S4.tif]

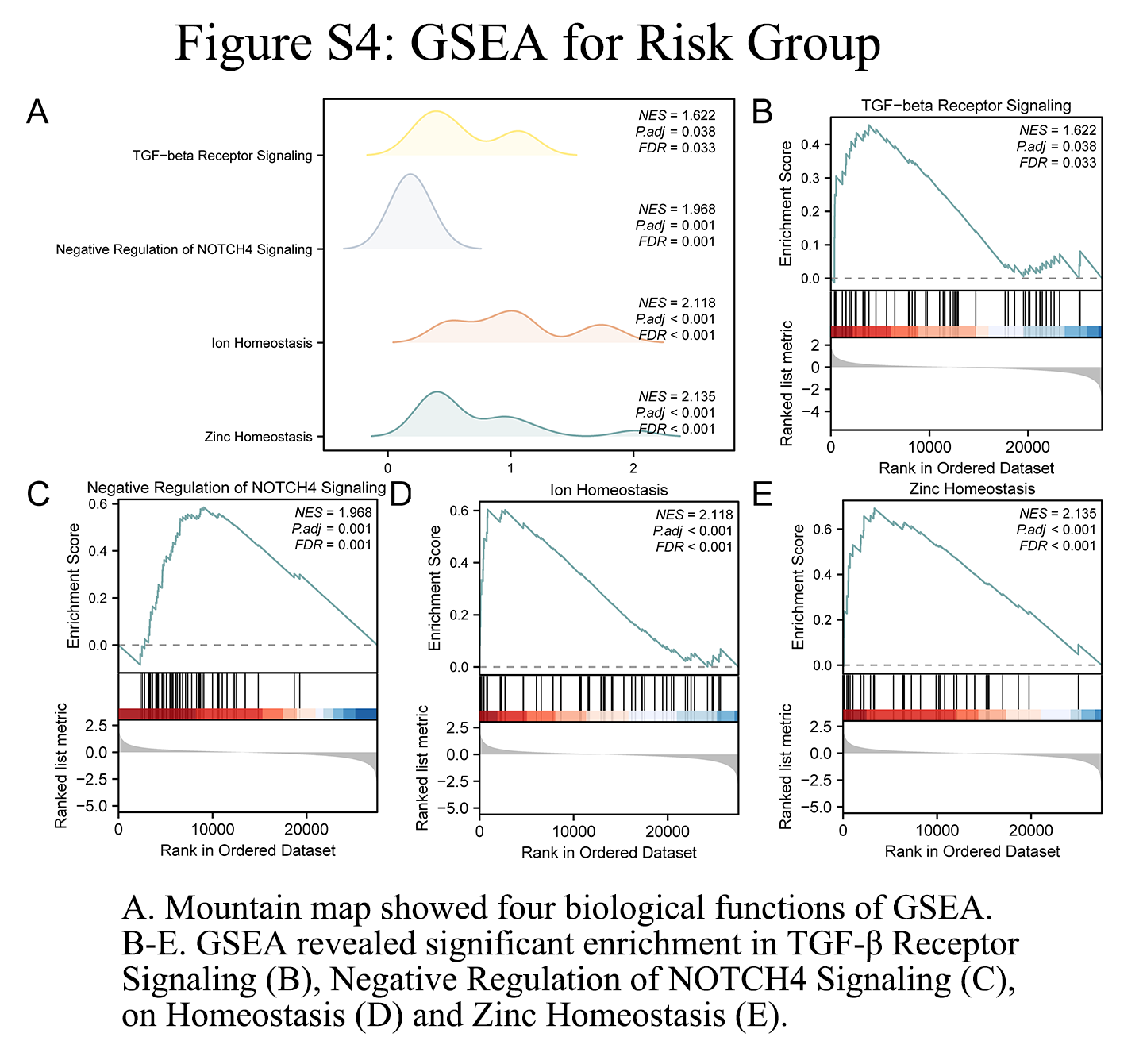

Supplement: Multimedia component 1 [file mmc1.zip › Supplementary Files/Figure S5.tif]

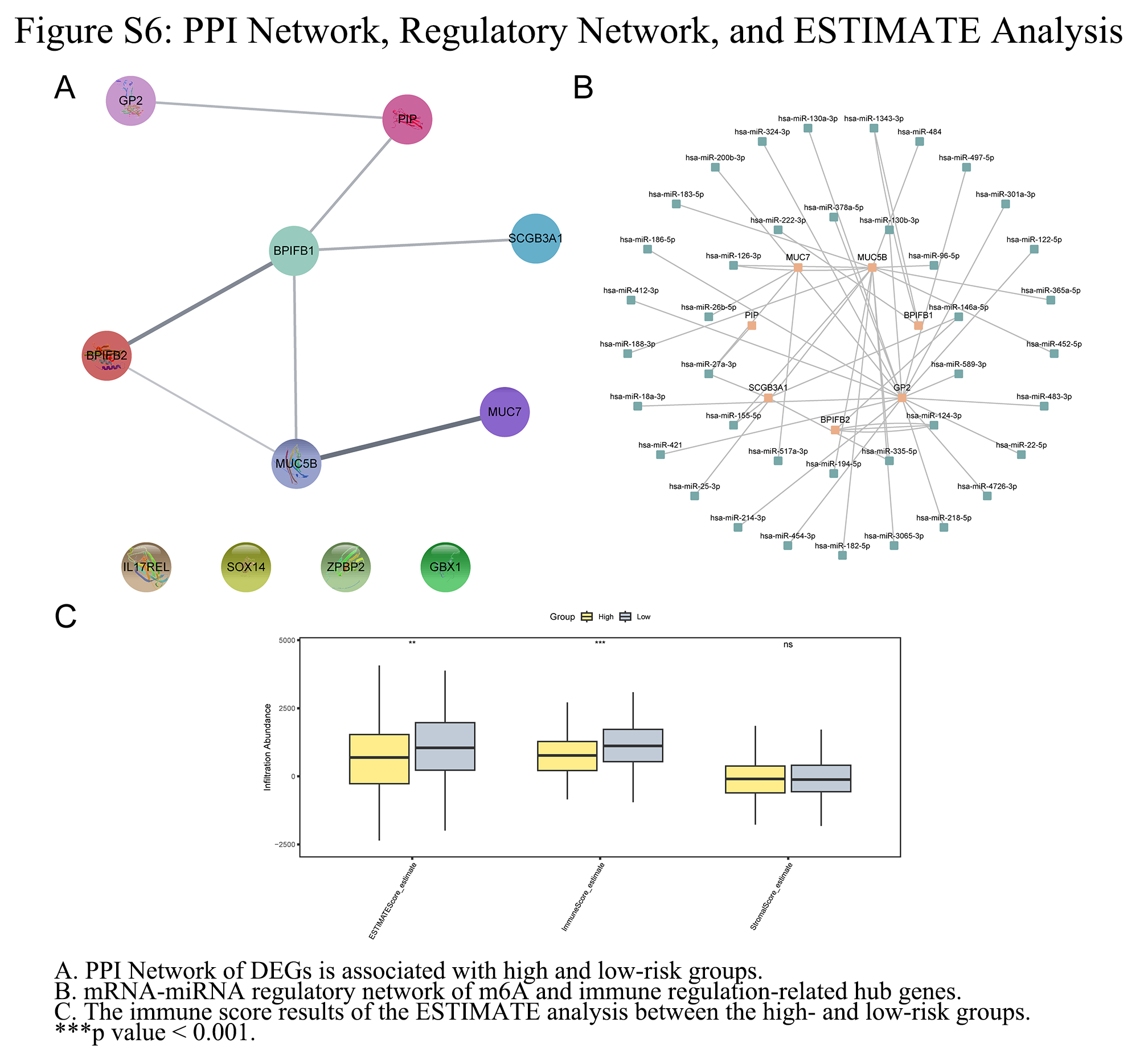

Supplement: Multimedia component 1 [file mmc1.zip › Supplementary Files/Figure S6.tif]
